# Prevalence and demographic, substance use, and mental health correlates of fasting among U.S. college students

**DOI:** 10.1186/s40337-021-00443-3

**Published:** 2021-07-21

**Authors:** Kyle T. Ganson, Rachel F. Rodgers, Stuart B. Murray, Jason M. Nagata

**Affiliations:** 1 Factor-Inwentash Faculty of Social Work, University of Toronto, Toronto, ON Canada; 2 APPEAR, Department of Applied Psychology, Northeastern University, Boston, MA USA; 3 Department of Psychiatric Emergency & Acute Care, Lapeyronie Hospital, CHRU Montpellier, Montpellier, France; 4 Department of Psychiatry and the Behavioral Sciences, University of Southern California, Los Angeles, CA USA; 5 Division of Adolescent and Young Adult Medicine, Department of Pediatrics, University of California, 550 16th Street, Box 0110, San Francisco, San Francisco, CA 94158 USA

**Keywords:** Fasting, Substance use, Mental health symptoms, Eating disorders, College students

## Abstract

**Background:**

Fasting is an unhealthy behavior that has been frequently used as part of weight loss attempts. To date, little research has been conducted to determine the prevalence and substance use and mental health correlates of fasting among college students. Therefore, the aim of this study was to estimate the prevalence and associations between any (≥ 1 time) and regular (≥ 13 times) occurrences of fasting in the past 4 weeks and substance use and mental health correlates among a large sample of college students from 2016 to 2020.

**Methods:**

Data from four academic survey years (2016–2020; *N* = 8255) of the national (USA) Healthy Minds Study were analyzed. Unadjusted prevalence of any and regular fasting by survey year and gender was estimated. Multiple logistic regression analyses were conducted to estimate the associations between any and regular fasting and the demographic (age, body mass index, race/ethnicity, sexual orientation, highest parental education), substance use (cigarette use, marijuana use, other illicit drug use, alcohol use), and mental health (depression, anxiety, eating disorder symptoms, suicidal ideation, non-suicidal self-injury) correlates.

**Results:**

Any fasting in the past 4 weeks was common among both men (14.77%) and women (18.12%) and significantly increased from 2016 (10.30%) to 2020 (19.81%) only among men. Regular fasting significantly increased among both men and women from 2016 (men: 1.46%; women: 1.79%) to 2020 (men: 3.53%; women: 6.19%). Among men and women, both any and regular fasting in the past 4 weeks were associated with higher odds of all mental health symptoms, including a positive depression, anxiety, and eating disorder screen, suicidal ideation, and non-suicidal self-injury. Among women, but not men, any and regular fasting in the past 4 weeks were associated with higher odds of marijuana use and other illicit drug use (e.g., cocaine, ecstasy).

**Conclusions:**

The results from this study underscore both the high and increasing prevalence of fasting among a national sample of college students, as well as the substance use and mental health symptoms associated with this behavior. Healthcare professionals both on and off campus should consider screening for fasting behaviors among college students and provide appropriate intervention when needed.

**Supplementary Information:**

The online version contains supplementary material available at 10.1186/s40337-021-00443-3.

## Plain english summary

Fasting has recently garnered public attention given its purported benefits, including weight loss and improved physical functioning. To date, little research has been conducted to determine the prevalence of this behavior among college students, as well as what demographic characteristics, substance use behaviors, and mental health symptoms are associated with this behavior. Results from a sample of over 8000 college students from four survey years of the Healthy Minds Study show that fasting is common among college men and women and has increased in prevalence from 2016 to 2020. Fasting is associated with indicators of poor mental health including depression, anxiety, eating disorder symptoms, suicidal ideation, and non-suicidal self-injury (e.g., cutting, burning), among both college men and women. Fasting is associated with substance use behaviors, including marijuana use and other illicit drug use (e.g., cocaine, ecstasy), among college women. These results highlight the adverse correlates of this common weight loss and compensatory behavior among college students.

**Supplementary Information:**

The online version contains supplementary material available at 10.1186/s40337-021-00443-3.

## Introduction

Fasting, the process of abstaining from food intake for extended periods of time, has grown in popularity over the last several years. The process of fasting, which can include alternate day fasting, periodic fasting, and time-restricted feeding, has been shown to have physiological consequences and elicit changes to the metabolic, cellular, and hormonal processes of the body [1–3]. While primarily used as a mechanism for weight loss or the modification of one’s body shape [1], some research has pointed to non-weight-related health benefits of fasting, such as improved physical and mental performance [2, 3], which may also explain its popularity. To date, research regarding the effects of fasting has primarily focused on testing whether fasting is an effective weight loss mechanism [4, 5], as well as identifying the potential benefits to the brain [6], and for reducing cancer [7, 8], cardiovascular disease, and diabetes [9, 10]. This existing research has left little known about the adverse correlates of this behavior.

Despite the interest in their potential health benefits, fasting, caloric restriction, and dietary restraint behaviors are associated with psychopathology, including suicidal ideation and suicide attempts [11, 12], non-suicidal self-injury [13, 14], and anxiety disorder and symptoms [15], as well as clinical and sub-clinical eating disorder symptoms. For example, the cyclical nature of fasting and as a period of unsustainably restricted eating may reduce cognitive control and promote binge eating behaviors [16, 17], and among both college women and men, dietary restraint was shown to be a primary predictor of eating pathology [18]. However, it is important to note that, while commonly used as a method to modify body shape and weight, fasting behaviors may be used by individuals pursuing different appearance or weight related goals, which may be differentially related to psychopathology. For instance, fasting may be used to pursue weight loss or to compensate for binge eating [19], or, more recently, fasting may be linked to growing trends promoting biohacking approaches to altering the body to improve physical and mental performance [20, 21]. Previous work has shown that, in particular, appearance goals may differ according to gender [22]. Thus, delineating the correlates of fasting among college men and women separately may offer important insights into the respective risk profiles.

To date, little research has been conducted to investigate fasting and its associations with substance use and poor mental health symptoms among college students. However, this age group is particularly vulnerable to engaging in fasting, as well as using substances and experiencing poor mental health symptoms. For example, eating disorders [23, 24], dieting, and disordered eating [25] regularly peak in later adolescence and young adulthood. Similarly, body dissatisfaction, a major driver of fasting and related disordered eating, is common among college age young adults [26]. College age young adults are also particularly susceptible to poor mental health, including depression, anxiety, suicidal ideation, and non-suicidal self-injury [27], as well as have increased access to alcohol and illicit drugs thus leading to substance use [28, 29]. Moreover, it is well established that eating disorders and disordered eating behaviors are connected with comorbid substance use and poor mental health [30–33]. The potential associations between fasting and substance use and poor mental health symptoms may be driven by the overlapping emotion regulation difficulties spanning these behaviors [34–36]. The combination of these factors indicates that further research is warranted among this age group to understand the connections between fasting and substance use and symptoms of poor mental health. Therefore, the aims of this study were, first, to estimate the prevalence and demographic correlates of fasting from 2016 to 2020, and second, to estimate the associations between fasting and several measures of substance use behaviors, including cigarette use, marijuana use, other illicit drug use, and alcohol use, and symptoms of poor mental health, including depression, anxiety, eating disorder behaviors, suicidal ideation, and non-suicidal self-injury, among a large, national sample of college students. While there are likely bi-directional relationships between fasting and substance use and poor mental health, we focus specifically on whether fasting, as the independent variable, predicts substance use behaviors and poor mental health. This decision was made a priori given that fasting appears to be increasingly prevalent for purposes of weight loss and body shape control, as well as for the purpose of improving physical and mental performance [1–3]. Given the previously reviewed literature, we hypothesized that prevalence of fasting will increase from 2016 to 2020 and will be associated with all indicators of substance use and poor mental health among both college men and women.

## Methods

We analyzed pooled data from four academic survey years (2016–2020; *N* = 8255) of the national (USA) Healthy Minds Study (HMS) [37]. HMS is an annual, repeat, cross-sectional study of several biological, psychological, and social health domains, as well as service utilization, among college undergraduate and graduate student participants. Colleges and universities voluntarily elect to participate in HMS and participants complete three standard modules, along with additional elective modules selected by their institution. At institutions with ≥ 4000 students, 4000 students are randomly recruited to participate in the survey; at institutions with < 4000 students, all students are recruited. To be eligible to participate, students must be at least 18 years old. Students are invited to participate via email and the survey is administered online via Qualtrics. Students are incentivized to participate using a drawing for Amazon gift cards. HMS is conducted out of the University of Michigan and Boston University, is approved by the Health Sciences and Behavioral Sciences Institutional Review Board at the University of Michigan, and informed consent was obtained from all participants [37]. This study was exempt from research ethics board approval given that HMS is publicly available and relies on secondary use of anonymous data.

### Measures

Fasting was measured using the question, “Over the past 4 weeks (28 days), how many times have you fasted (intentionally not eaten anything at all for at least 8 waking hours)?” This question is part of the widely used Eating Disorder Examination Questionnaire (EDE-Q) [38]. Given the significant positive skewness (7.5) and kurtosis (126.6) of the fasting variable, and to align with previously identified clinical cut-off points, responses were dichotomized into *any* (zero [0] times and one [1] or more times) and *regular* (0–12 times and 13 or more times) fasting, as has been done in prior research [39–41].

Cigarette use was measured using the question: “Over the past 30 days, about how many cigarettes did you smoke per day?” This variable was converted into a dichotomous variable with participants who reported zero (0) cigarette use and those who reported any cigarette use [42, 43].

Illicit drug use was measured using the question, “Over the past 30 days, have you used any of the following drugs?” Potential response options included: “Marijuana;” “Cocaine (any form, including crack, powder, and freebase);” “Heroin”… Two dichotomous variables were created to investigate illicit drug use: Marijuana use and other illicit drug use. Marijuana use included those who reported any marijuana use and those who reported none. Other illicit drug use included those who reported any illicit drug use (minus marijuana use) and those who reported none.

Alcohol use was measured using the question: “Over the past 2 weeks, did you drink alcohol?” A dichotomous “yes” or “no” response was available.

Depression was measured using the Patient Health Questionnaire-9 (PHQ-9). This screening tool is based on the nine *Diagnostic and Statistical Manual for Mental Disorders* (*DSM*)-*IV* criteria for a major depressive episode and includes questions such as “Over the last 2 weeks, how often have you been bothered by any of the following problems? Little interest or pleasure in doing things; Feeling down, depressed or hopeless; Trouble falling or staying asleep, or sleeping too much” [44, 45]. Responses for each question include, “Not at all;” “Several days;” “More than half the days;” and “Nearly every day.” Scores range from 0 to 27. This variable was converted into a dichotomous variable with participants who screened negative (scores 0–9) and who screened positive (scores 10-27) for depression [44, 45].

Anxiety was measured using the Generalized Anxiety Disorder 7-Item (GAD-7). This screening tool was developed to reflect all components of the *Diagnostic and Statistical Manual for Mental Disorders* (*DSM*)-*IV* criteria for generalized anxiety disorder and includes questions such as “Over the last 2 weeks, how often have you been bothered by the following problems? Feeling nervous, anxious or on edge; Not being able to stop or control worrying; Worrying too much about different things” [46]. Responses for each question include: “Not at all;” “Several days;” “More than half the days;” and “Nearly every day.” Scores range from 0 to 21. This variable was converted into a dichotomous variable with participants who screened negative (scores 0–9) and those who screened positive (scores 10-21) for anxiety [46].

Eating disorders were measured using the 5-item SCOFF questionnaire [47]. The items include “Do you ever make yourself sick because you feel uncomfortably full? Do you worry that you have lost control over how much you eat? Have you recently lost more than 15 pounds in a 3-month period? Do you believe yourself to be fat when others say you are too thin? Would you say that food dominates your life?” A positive eating disorder screen was indicated by a score of two (2) or more “yes” responses [47, 48]. This cut off score has been shown to have a sensitivity of 70–100% and specificity of 73–94% for anorexia nervosa, bulimia nervosa, and binge-eating disorder [47–49].

Suicidal ideation was measured using the question, “In the past year, did you ever seriously think about attempting suicide?” A dichotomous “yes” or “no” response was available. This item has been used in prior research [27, 50].

Non-suicidal self-injury (NSSI) was measured using the question: “In the past year, have you ever done any of the following intentionally?” Potential responses include: “Cut myself;” “Burned myself;” “Punched or banged myself;” “Scratched myself;” “Pulled my hair;” “Bit myself;” “Interfered with a wound healing;” “Carved words of symbols into my skin;” “Rubbed sharp objects into my skin;” “Punched or banged an object to hurt myself;” and “Other.” This variable was converted into a dichotomous variable with participants who reported any NSSI and those who reported none as has been done in prior research [27, 42, 51].

### Demographic variables

Demographic variables included: self-reported age (range 18 to 79 years; 93.3% ≤ 30 years), race/ethnicity (White or Caucasian, non-Hispanic, non-Arab; Black or African American, non-Hispanic; Hispanic/Latino/a; Asian or Asian American; Arab/Middle Eastern or Arab American; American Indian, Alaskan Native, Native Hawaiian or Pacific Islander; other race/ethnicity; more than 1 race/ethnicity), sexual orientation (heterosexual; gay or lesbian; queer, questioning, or other), and highest parent education. Body mass index (BMI) was calculated based on self-reported height and weight (kg/m^2^). In accordance with prior research on fasting and eating disorder behaviors [11, 15, 19, 52], these demographic variables were adjusted for in the aim two analyses.

### Statistical analysis

Descriptive analyses were conducted to characterize the sample. To determine our first aim, unadjusted prevalence of *any* and *regular* fasting by survey year were estimated among college men and women (Pearson chi-square tests). Multivariable logistic regression analyses were conducted to estimate the associations between the demographic variables and survey year as the independent variables, and *any* and *regular* fasting as the dependent variables, among college men and women. To determine our second aim, we pooled the survey years given the differing number of participants at each survey year (2016/2017: 1435; 2017/2018: 4352; 2018/2019: 2026; 2019/2020: 442), which increased statical power. Multiple logistic regression analyses were conducted to estimate the associations between *any* and *regular* fasting as the independent variables, and the substance use behaviors (cigarette use, marijuana use, other illicit drug use, alcohol use) and poor mental health symptoms (depression screen, anxiety screen, eating disorder screen, suicidal ideation, NSSI) as the dependent variables, while adjusting for the demographic variables. We also adjusted for survey year to account for any potential influence a single year may have had on the results. All analyses were stratified by gender given the potential differences in the purpose of fasting [19, 21]. Statistical analyses included preconstructed sample weighting to adjust for nonresponse bias. Sample weights were constructed based on gender, race/ethnicity, academic level, and grade point average. Participants with underrepresented demographic characteristics are assigned greater sample weights [37]. Additional supplemental analyses (results stratified by year and BMI) are displayed in the online supplement. All analyses were conducted in 2021 using Stata 15.1 [53].

## Results

Among the diverse sample of college student participants (Table 1), 67.59% (*n* = 5580) were women. The mean age and BMI for men were 22.96 (SE ± 0.17) years and 24.72 (SE ± 0.14), respectively, while the mean age and BMI for women were 22.31 (SE ± 0.12) years and 24.28 (SE ± 0.09), respectively. Roughly two thirds of both men (65.08%) and women (66.86%) in the sample identified as White or Caucasian, non-Hispanic, non-Arab. The majority of both men and women identified as heterosexual and had a highest parental education of some college or more. Women (18.12%; *p* < 0.001) had slightly higher prevalence of *any* fasting in the past 4 weeks, while there was no statistical difference in prevalence of *regular* fasting in the past 4 weeks among men (3.14%) and women (2.81%).

**Table 1:** Demographic Characteristics and Descriptive Statistics of College Student Participants from the 2016–2020 Healthy Minds Study (*N* = 8255)

	Men (*n* = 2675)	Women (*n* = 5580)
	Mean ± SE/%	Mean ± SE/%
Demographic characteristics		
Age	22.96 ± 0.17	22.31 ± 0.12
Body mass index (kg/m^2^)	24.72 ± 0.14	24.28 ± 0.09
Race/ethnicity		
White or Caucasian, non-Hispanic, non-Arab	65.08	66.86
Black or African American, non-Hispanic	3.52	5.82
Hispanic/Latino/a	5.13	5.20
Asian or Asian American	13.10	10.73
Arab/Middle Eastern or Arab American	1.58	0.66
American Indian, Alaskan Native, Native Hawaiian or Pacific Islander	0.11	0.31
Other race/ethnicity	1.73	0.58
More than 1 race/ethnicity	9.74	9.83
Sexual orientation		
Heterosexual	86.04	80.87
Gay or lesbian	6.52	3.50
Bisexual	3.94	10.86
Queer, questioning, or other	3.50	4.78
Highest parental education		
High school degree or less	10.19	8.69
Some college or more	89.81	91.31
Substance use correlates		
Any cigarette use, past 30 days	10.88	6.45
Marijuana use, past 30 days	24.72	20.62
Other illicit drug use, past 30 days	8.21	5.11
Alcohol use, past 2 weeks	65.14	66.92
Mental health correlates		
Positive depression screen, PHQ-9	21.87	26.91
Positive anxiety screen, GAD-7	15.67	25.36
Positive eating disorder screen, SCOFF	13.54	26.09
Suicidal ideation, past 12 months	8.99	9.33
Any non-suicidal self-injury, past 12 months	15.60	20.34
Any fasting (≥ 1 times), past 4 weeks	14.77	18.12
Regular fasting (≥ 13 times), past 4 weeks	3.14	2.81

Preconstructed nonresponse sample weighting was applied to all analyses

### Aim one results: the prevalence and demographic correlates of fasting from 2016 to 2020

**Fig. 1:**
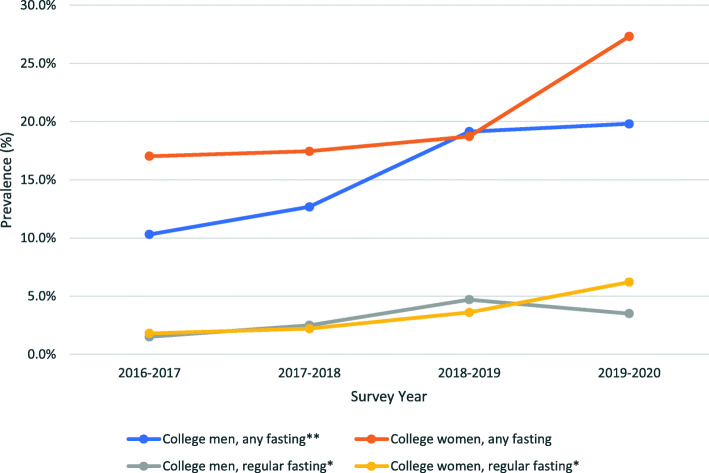
Prevalence (%) of Any (≥ 1 Times) and Regular (≥ 13 Times) Fasting in the Past Four Weeks by Gender and Survey Year. *p < 0.01. **p < 0.001

Results related to our first aim showed that, among both men and women, prevalence of *any* fasting in the past 4 weeks increased from 2016 to 2020 and was highest for both men (19.81%) and women (27.32%) in the 2019–2020 survey year. *Any* fasting increased 9.5 percentage points for men and 10.3 percentage points for women from 2016 to 2020. This increase was particularly significant among men (*p* < 0.001). Among both men and women, prevalence of *regular* fasting in the past 4 weeks significantly increased (*p* < 0.01) from 2016 to 2020 and was highest for men (4.66%) in the 2018–2019 survey year and women (6.19%) in the 2019–2020 survey year. *Regular* fasting increased 2.0 percentage points for men and 4.4 percentage points for women (Fig. 1).

Results from multivariable logistic regressions analyses revealed significant associations between the demographic variables and fasting. Regarding *any* fasting (Table 2), among both men and women, higher BMI (men: adjusted odds ratio [AOR] 1.04, 95% confidence interval [CI] 1.01–1.07; women: AOR 1.03, 95% CI 1.02–1.05) was associated with greater odds of *any* fasting in the past 4 weeks. Among both men and women, bisexual (men: AOR 2.64, 95% CI 1.58–4.40; women: AOR 1.65, 95% CI 1.27–2.15) and queer, questioning, or other (men: AOR 2.50, 95% CI 1.26–4.95; women: AOR 1.71, 95% CI 1.21–2.42) sexual orientation was associated with greater odds of *any* fasting in the past 4 weeks. Among men, gay sexual orientation (AOR 2.52, 95% CI 1.44–4.42) was associated with greater odds of *any* fasting in the past 4 weeks. Among men, those who identified as multi-racial (AOR 1.77, 95% CI 1.16–2.69) had greater odds of *any* fasting in the past 4 weeks. Among women, younger age (AOR 0.95, 95% CI 0.93–0.98) was associated with *any* fasting in the past 4 weeks. Among men, the odds of *any* fasting in the past 4 weeks increased from 2016 to 2020 (AOR 1.45, 95% CI 1.21–1.74).

**Table 2:** Associations between Demographic Correlates and Survey Year and Any Fasting (≥ 1 Times) in the Past Four Weeks by Gender

	Men	*p*	Women	*p*
	AOR (95% CI)		AOR (95% CI)	
Age	0.98 (0.95–1.00)	0.126	**0.95 (0.93–0.98)**	**< 0.001**
Body mass index (kg/m^2^)	**1.04 (1.01–1.07)**	**0.004**	**1.03 (1.02–1.05)**	**< 0.001**
Race/ethnicity				
White or Caucasian, non-Hispanic, non-Arab	Ref.	Ref.	Ref.	Ref.
Black or African American, non-Hispanic	1.34 (0.67–2.65)	0.403	0.78 (0.52–1.18)	0.249
Hispanic/Latino/a	1.08 (0.60–1.92)	0.794	1.06 (0.72–1.56)	0.782
Asian or Asian American	0.78 (0.51–1.18)	0.241	0.96 (0.73–1.25)	0.748
American Indian, Alaskan Native, Native Hawaiian or Pacific Islander	–	–	2.25 (0.38–13.35)	0.372
Arab/Middle Eastern or Arab American	1.42 (0.56–3.58)	0.456	0.98 (0.38–2.52)	0.976
Other race/ethnicity	1.70 (0.70–4.13)	0.241	1.39 (0.53–3.68)	0.504
More than 1 race/ethnicity	**1.77 (1.16–2.69)**	**0.007**	1.13 (0.86–1.47)	0.381
Sexual orientation				
Heterosexual	Ref.	Ref.	Ref.	Ref.
Gay or lesbian	**2.52 (1.44–4.42)**	**0.001**	1.28 (0.85–1.94)	0.238
Bisexual	**2.64 (1.58–4.40)**	**< 0.001**	**1.65 (1.27–2.15)**	**< 0.001**
Queer, questioning, or other	**2.50 (1.26–4.95)**	**0.009**	**1.71 (1.21–2.42)**	**0.002**
Parental education				
High school degree or less	Ref.	Ref.	Ref.	Ref.
Some college or more	0.68 (0.40–1.15)	0.154	0.84 (0.59–1.19)	0.335
Survey year	**1.45 (1.21–1.74)**	**< 0.001**	1.05 (0.94–1.18)	0.350

Preconstructed nonresponse sample weighting was applied to all analyses

**Boldface** indicates statistical significance (*p* < 0.05)

*AOR* Adjusted odds ratio, *CI* Confidence interval

Regarding *regular* fasting (Table 3), among both men and women, the odds of *regular* fasting in the past 4 weeks increased from 2016 to 2020 (men: AOR 1.64, 95% CI 1.16–2.31; women: AOR 1.39, 95% CI 1.04–1.86). Among women, younger age (AOR 0.95, 95% CI 0.92–0.99) and those who identified as Hispanic/Latina (AOR 0.33, 95% CI 0.14–0.79) had lower odds of *regular* fasting in the past 4 weeks. Among women, bisexual (AOR 1.85, 95% CI 1.00–3.41) and queer, questioning, or other (AOR 2.76, 95% CI 1.24–6.15) sexual orientation had higher odds of *regular* fasting in the past 4 weeks.

**Table 3:** Associations between Demographic Correlates and Survey Year and Regular Fasting (≥ 13 Times) in the Past Four Weeks by Gender

	Men	*p*	Women	*p*
	AOR (95% CI)		AOR (95% CI)	
Age	1.02 (0.99–1.05)	0.244	**0.95 (0.92–0.99)**	**0.029**
Body mass index (kg/m^2^)	1.03 (0.99–1.07)	0.118	1.02 (0.99–1.06)	0.179
Race/ethnicity				
White or Caucasian, non-Hispanic, non-Arab	Ref.	Ref.	Ref.	Ref.
Black or African American, non-Hispanic	0.59 (0.13–2.59)	0.485	0.71 (0.26–1.97)	0.514
Hispanic/Latino/a	1.68 (0.56–4.96)	0.350	**0.33 (0.14–0.79)**	**0.013**
Asian or Asian American	0.76 (0.33–1.76)	0.523	0.78 (0.37–1.61)	0.502
American Indian, Alaskan Native, Native Hawaiian or Pacific Islander	–	–	–	–
Arab/Middle Eastern or Arab American	1.97 (0.46–8.37)	0.359	–	–
Other race/ethnicity	1.36 (0.33–5.67)	0.671	1.55 (0.19–12.41)	0.677
More than 1 race/ethnicity	1.40 (0.56–3.51)	0.467	1.39 (0.76–2.54)	0.289
Sexual orientation				
Heterosexual	Ref.	Ref.	Ref.	Ref.
Gay or lesbian	1.53 (0.43–5.39)	0.510	1.57 (0.62–3.95)	0.336
Bisexual	1.83 (0.64–5.27)	0.262	**1.85 (1.00–3.41)**	**0.049**
Queer, questioning, or other	1.31 (0.41–4.17)	0.648	**2.76 (1.24–6.15)**	**0.013**
Parental education				
High school degree or less	Ref.	Ref.	Ref.	Ref.
Some college or more	1.87 (0.75–4.62)	0.177	**0.39 (0.20–0.79)**	**0.009**
Survey year	**1.64 (1.16–2.31)**	**0.005**	**1.39 (1.04–1.86)**	**0.026**

Preconstructed nonresponse sample weighting was applied to all analyses

**Boldface** indicates statistical significance (*p* < 0.05)

*AOR* Adjusted odds ratio, *CI* Confidence interval

### Aim two results: associations between fasting, substance use behaviors, and symptoms of poor mental health

Results from logistic regression analyses revealed significant associations between *any* and *regular* fasting and substance use behaviors and poor mental health symptoms (Table 4), although these patterns varied according to frequency of fasting and gender. Regarding reports of *any* fasting over the past 4 weeks, among men, *any* fasting was significantly associated with greater odds of a positive depression screen (AOR 3.40, 95% CI 2.53–4.56), a positive anxiety screen (AOR 2.73, 95% CI 1.98–3.78), a positive eating disorder screen (AOR 4.64, 95% CI 3.35–6.43), self-reported suicidal ideation in the past 12 months (AOR 2.44, 95% CI 1.62–3.68), and self-reported NSSI in the past 12 months (AOR 2.53, 95% CI 1.84–3.50) while adjusting for the demographic variables and survey year. *Any* fasting in the past 4 weeks was significantly associated with lower odds of alcohol use in the past 4 weeks (AOR 0.72 95% CI 0.54–0.96) while adjusting for the demographic variables and survey year. There were no significant associations between *any* fasting and any cigarette use in the past 30 days, any marijuana use in the past 30 days, and any other illicit drug use in the past 30 days.

**Table 4:** Associations between Any Fasting (≥ 1 Times) and Regular Fasting (≥ 13 Times) in the Past Four Weeks and Substance Use and Mental Health Correlates by Gender among Participants from the Healthy Minds Study

	Men		Women	
Any fasting	AOR^a^ (95% CI)	*p*	AOR^a^ (95% CI)	*p*
Any cigarette use, past 30 days	1.36 (0.90–2.06)	0.142	**1.57 (1.15–2.15)**	**0.005**
Marijuana use, past 30 days	1.34 (0.99–1.83)	0.060	**1.92 (1.58–2.34)**	**< 0.001**
Other illicit drug use, past 30 days	1.45 (0.90–2.31)	0.123	**2.71 (1.88–3.90)**	**< 0.001**
Alcohol use, past 2 weeks	**0.72 (0.54–0.96)**	**0.027**	1.14 (0.94–1.37)	0.179
Positive depression screen, PHQ-9	**3.40 (2.53–4.56)**	**< 0.001**	**3.86 (3.20–4.65)**	**< 0.001**
Positive anxiety screen, GAD-7	**2.73 (1.98–3.78)**	**< 0.001**	**2.58 (2.14–3.11)**	**< 0.001**
Positive eating disorder screen, SCOFF	**4.64 (3.35–6.43)**	**< 0.001**	**4.86 (4.02–5.87)**	**< 0.001**
Suicidal ideation, past 12 months	**2.44 (1.62–3.68)**	**< 0.001**	**2.72 (2.11–3.50)**	**< 0.001**
Any non-suicidal self-injury, past 12 months	**2.53 (1.84–3.50)**	**< 0.001**	**2.63 (2.15–3.22)**	**< 0.001**
	Men		Women	
Regular fasting	AOR^a^ (95% CI)	*p*	AOR^a^ (95% CI)	*p*
Any cigarette use, past 30 days	0.68 (0.23–1.98)	0.480	1.58 (0.72–3.43)	0.250
Marijuana use, past 30 days	1.52 (0.81–2.84)	0.195	**2.14 (1.34–3.41)**	**0.001**
Other illicit drug use, past 30 days	0.80 (0.30–2.12)	0.657	**4.86 (2.34–10.10)**	**< 0.001**
Alcohol use, past 2 weeks	0.86 (0.47–1.55)	0.613	1.15 (0.72–1.84)	0.565
Positive depression screen, PHQ-9	**3.70 (2.07–6.62)**	**< 0.001**	**5.19 (3.22–8.37)**	**< 0.001**
Positive anxiety screen, GAD-7	**4.32 (2.32–8.04)**	**< 0.001**	**4.27 (2.67–6.84)**	**< 0.001**
Positive eating disorder screen, SCOFF	**7.02 (3.77–13.09)**	**< 0.001**	**6.38 (3.85–10.57)**	**< 0.001**
Suicidal ideation, past 12 months	**3.00 (1.43–6.26)**	**0.004**	**3.67 (2.24–6.00)**	**< 0.001**
Any non-suicidal self-injury, past 12 months	**3.09 (1.65–5.80)**	**< 0.001**	**2.99 (1.81–4.95)**	**< 0.001**

Preconstructed nonresponse sample weighting was applied to all analyses

**Boldface** indicates statistical significance (*p* < 0.05)

*AOR* Adjusted odds ratio, *CI* Confidence interval

^a^Adjusted for age, body mass index (kg/m^2^), race/ethnicity, sexual orientation, highest parent education, and survey year

Among women, *any* fasting in the past 4 weeks was significantly associated with greater odds of any cigarette use in the past 30 days (AOR 1.57, 95% CI 1.15–2.15), any marijuana use in the past 30 days (AOR 1.92, 95% CI 1.58–2.34), any other illicit drug use in the past 30 days (AOR 2.71, 95% CI 1.88–2.34), a positive depression screen (AOR 3.86, 95% CI 3.20–4.65), a positive anxiety screen (AOR 2.58, 95% CI 2.14–3.11), a positive eating disorder screen (AOR 4.86, 95% CI 4.02–5.87), self-reported suicidal ideation in the past 12 months (AOR 2.72, 95% CI 2.11–3.50), and self-reported NSSI in the past 12 months (AOR 2.63, 95% CI 2.15–3.22) while adjusting for the demographic variables and survey year. There were no significant associations between *any* fasting and alcohol use in the past 4 weeks.

Regarding *regular* fasting in the past 4 weeks, among men, *regular* fasting was significantly associated with greater odds of a positive depression screen (AOR 3.70, 95% CI 2.07–6.62), a positive anxiety screen (AOR 4.32, 95% CI 2.32–8.04), a positive eating disorder screen (AOR 7.02, 95% CI 3.77–13.09), self-reported suicidal ideation in the past 12 months (AOR 3.00, 95% CI 1.43–6.26), and self-reported NSSI in the past 12 months (AOR 3.09, 95% CI 1.65–5.80) while adjusting for the demographic variables and survey year. There were no significant associations between *regular* fasting and the substance use measures.

Among women, *regular* fasting in the past 4 weeks was significantly associated with greater odds of any marijuana use in the past 30 days (AOR 2.14, 95% CI 1.34–3.41), any other illicit drug use in the past 30 days (AOR 4.86, 95% CI 2.34–10.10), a positive depression screen (AOR 5.19, 95% CI 3.22–8.37), a positive anxiety screen (AOR 4.27, 95% CI 2.67–6.84), a positive eating disorder screen (AOR 6.38, 95% CI 3.85–10.57), self-reported suicidal ideation in the past 12 months (AOR 3.67, 95% CI 2.24–6.00), and self-reported NSSI in the past 12 months (AOR 2.99, 95% CI 1.81–4.95) while adjusting for the demographic variables and survey year. There were no significant associations between *regular* fasting and any cigarette use in the past 30 days and alcohol use in the past 2 weeks.

Overall, findings from the supplemental analyses were mostly similar with few differences when stratified by BMI (see Supplement).

## Discussion

The aims of this study were to estimate the prevalence and demographic, substance use, and mental health correlates of fasting from 2016 to 2020 among a large, national sample of college men and women. The results showed that fasting has significantly increased from 2016 to 2020 among both men and women, with nearly 20% of men and 27% of women reporting fasting one or more times in the 2019–2020 survey year.

Results revealed unique demographic correlates of *any* and *regular* fasting among the sample. Among both men and women, higher BMI was associated with *any* fasting, which is not surprising given that a primary purpose of fasting is weight loss [1]; however, BMI was not associated with *regular* fasting. This finding my indicate that heightened risk for fasting is present among those with higher BMI solely when *any* fasting occurs, which may be due to the overall difficulty of maintaining fasting behaviors over many days within a four-week period. Among both men and women, sexual minority identity (e.g., gay or lesbian, bisexual, or queer, questioning or other) was associated with greater odds of *any* fasting, and greater odds of *regular* fasting was only among women. Prior research has shown that sexual minority young adults often engage in disordered eating behaviors at greater prevalence than their heterosexual peers, which may be interpreted in the context of minority stress theory [41, 54]. Importantly, the bisexual men and women had the strongest association with *any* fasting, as well as *regular* fasting among women. This may be further evidence of the detrimental effects of “biphobia” [55]. Among men, identifying as multi-racial was associated with higher odds of *any* fasting. This may be attributed to the intersectional identities of gender and race/ethnicity [56], as well as potentially unique appearance ideals [57]. Interestingly, among women, Hispanic/Latina identity was associated with lower odds of *regular* fasting, which may be evidence of stronger ethnic identity and lower eating disorder symptoms [57]. Additionally, among women, those with a highest parental education of some college or more was associated with lower odds of *regular* fasting. This finding, along with others related to educational attainment [58, 59] may elucidate educational and class differences of *regular* fasting; however, there is conflicting evidence of the impact of education, both parental and self, as it relates to eating disorders [60].

Our results showed that both *any* and *regular* fasting was strongly associated with substance use behaviors among women and symptoms of poor mental health among both men and women. Among men and women, both *any* and *regular* fasting were associated with higher odds of all of the poor mental health symptoms assessed. However, the strength of the associations between fasting and symptoms of poor mental health were greater among those who reported *regular* fasting. This highlights the potentially detrimental mental health associations of *regular* fasting among college students. Furthermore, *regular* fasting may be a behavioral indicator of poor mental health among college students. These results highlight that psychiatric comorbidities remain common alongside eating disorder behaviors [30, 61], and that men may experience similar or even greater levels of psychological impairment along with women [62, 63].

While, overall, the patterns of poor mental health symptoms presented similarities across men and women, there were stark differences among substance use correlates. Among women, those who reported *any* fasting had greater odds of any cigarette smoking, marijuana use, and other illicit drug use. Interestingly, cigarette smoking was no longer associated with fasting among women who reported *regular* fasting, as opposed to less frequent fasting. Conversely, *regular* fasting was associated with nearly two-fold greater odds of marijuana use and nearly five-fold greater odds of other illicit drug use among women. While cigarette smoking [64] may be used to augment weight loss efforts given their appetite suppressant qualities, it may be that illicit drugs (e.g., stimulants, cocaine, ecstasy) are more effective for weight loss and suppressing appetite [65–67]. Relatedly, marijuana use is common among college age young adults [29], and fasting may be a mechanism to compensate for additional caloric intake given the common effect of increased appetite while under the influence of marijuana. These results among women further highlight the cooccurrence of substance use and eating disorders [30, 68].

Interestingly, among men, there were no significant associations between *any* and *regular* fasting and any cigarette smoking, marijuana use, and other illicit drug use. In fact, among men, *any* fasting was associated with lower odds of alcohol use in the past 2 weeks. This may provide further evidence that fasting is a mechanism to improve psychological and physical performance [20, 21], as well as reducing body adiposity to improve muscularity and leanness [61, 69]. Thus, the use of substances would interfere with this intended outcome. Fasting has been shown to be a mechanism to compensate for the caloric intake from alcohol use [70, 71], which these results indicate may not be true among men. This is further supported by sensitivity analyses exploring *any* and *regular* fasting and binge-drinking, among both men and women, which resulted in similarly null findings.

The results from this study have several important implications. Despite some of the benefits of fasting, particularly while under the advisement of clinical professionals, portrayed in prior research [1–10], the results from this study should caution individuals from engaging in this behavior, specifically those without appropriate oversight and supervision from a medical professional. Furthermore, the cultural and social promotion of fasting as a positive and effective behavior should be reassessed and minimized.

The results from this study also point to several theoretical implications and areas for future research. In general, fasting aligns more with traditional conceptualizations of drive for thinness and eating disorders among women [19, 72] and occurs at greater prevalence among girls and women [73]. However, descriptively, women (18.12% vs. 14.77%) had higher prevalence of *any* fasting, while men (3.14% vs. 2.81%) had higher prevalence of *regular* fasting. This aligns with prior research showing that between 1.4–7.7% of boys and men, depending on age and timeframe, engage in fasting behaviors [73]. Furthermore, in this study, *regular* fasting was associated with over seven-fold greater odds of a positive eating disorder screen among men. These results indicate that traditional conceptualizations of fasting may be shifting, where, not only is the fasting behavior intended for optimizing performance [20, 21], it is also associated with traditional eating disorder symptomology among men. It seems clear that the presentation of eating disorders, disordered eating, and performance-enhancing behaviors are complex among men and further research is needed to further conceptualize these behaviors to better describe their etiology.

### Strengths and limitations

There are several important strengths to this study. These include the use of a national, large, and diverse sample of college men and women, as well as the use of several substance use and mental health measures. Despite these strengths, there are limitations to be noted. HMS is a cross-sectional survey, which limits the ability to draw inferences regarding causal relationships. This is particularly important given that it is highly likely that the relationships between fasting and substance use and poor mental health are bi-directional. Thus, future research is needed, particularly among a longitudinal cohort sample, to characterize the temporal relationships between these behaviors. Relatedly, given that HMS uses a module-based survey design, the number of participants who completed the module that included the fasting item differed from year to year (2016/2017: 1435; 2017/2018: 4352; 2018/2019: 2026; 2019/2020: 442), which may introduce selection bias. However, we adjusted for survey year in our analyses in an attempt to account for this potential bias. Furthermore, substantially more women than men were included in this study, which may impact generalizability of the findings. Survey items are based on self-report, which increases the potential for reporting bias; however, research has shown that young people are more willing to self-disclose sensitive information in web-based surveys [74–76]. Relatedly, the fasting, substance use, and mental health variables were dichotomized for analysis, which may have oversimplified these behaviors and symptoms and impacted the results. Future research is needed to explore the breadth of substance use behaviors and poor mental health symptoms, including frequency, dose, intensity, and timing to further characterize the associations found in this study. Despite adjusting for several confounders (e.g., age, race/ethnicity) that may influence the relationship between the variables under study, there is the potential for unmeasured confounders. We were unable to conduct analyses among transgender and gender non-conforming college students due to a lack of statistical power. Lastly, we were unable to ascertain from the data the intensity, purpose, motivations, and true duration of the fasting behavior among participants. Thus, further research is needed to among the different demographic groups to add to our findings and further describe when and how fasting leads to substance use and poor mental health.

## Conclusion

The purpose of this study was to determine the prevalence and demographic, substance use, and mental health correlates of fasting among college men and women. The results show that fasting is increasing among both men and women, with nearly one in five men and over one in four women reporting *any* fasting in the past 4 weeks. *Any* and *regular* fasting in the past 4 weeks were associated with key demographic correlates, including BMI, age, and sexual orientation. Furthermore, *any* and *regular* fasting in the past 4 weeks were associated with significantly greater odds of poor mental health symptoms among both college men and women and substance use behaviors among college women. Despite the purported benefits of fasting, as well as the social and cultural promotion in the media, there is cause for concern given these associations. Clinical professionals should screen for fasting and related psychopathology among young adults.

## Supplementary Information

**Additional file 1: Supplement Table 1**. Demographic Characteristics and Descriptive Statistics of College Student Participants from the 2016–2020 Healthy Minds Study (*N* = 8255) by Gender and Survey Year. **Supplement Table 2**. Associations between Demographic Correlates and Survey Year and Any Fasting (≥ 1 Times) in the Past Four Weeks by Gender and BMI. **Supplement Table 3**. Associations between Demographic Correlates and Survey Year and Regular Fasting (≥ 13 Times) in the Past Four Weeks by Gender and BMI. **Supplement Fig. 1**. Prevalence (%) of Any (≥ 1 Times) and Regular (≥ 13 Times) Fasting in the Past Four Weeks among Participants with Body Mass Index < 25, by Gender and Survey Year. **Supplement Fig. 2**. Prevalence (%) of Any (≥ 1 Times) and Regular (≥ 13 Times) Fasting in the Past Four Weeks among Participants with Body Mass Index ≥ 25, by Gender and Survey Year. **Supplement Table 4**. Associations between Any Fasting (≥ 1 Times) and Regular Fasting (≥ 13 Times) in the Past Four Weeks and Substance Use and Mental Health Correlates by Gender and BMI among Participants from the Healthy Minds Study.

## Data Availability

The Healthy Minds Study is available to researchers. Please visit http://healthymindsnetwork.org for more information.

## References

[1] Freire R (2020). Scientific evidence of diets for weight loss: different macronutrient composition, intermittent fasting, and popular diets. Nutrition [internet].

[2] Anton SD, Moehl K, Donahoo WT, Marosi K, Lee SA, Mainous AG (2018). Flipping the metabolic switch: understanding and applying the health benefits of fasting. Obesity..

[3] de Cabo R, Mattson MP (2019). Effects of intermittent fasting on health, aging, and disease. N Engl J Med.

[4] Lowe DA, Wu N, Rohdin-Bibby L, Moore AH, Kelly N, Liu YE (2020). Effects of time-restricted eating on weight loss and other metabolic parameters in women and men with overweight and obesity: the TREAT randomized clinical trial. JAMA Intern Med.

[5] Cioffi I, Evangelista A, Ponzo V, Ciccone G, Soldati L, Santarpia L (2018). Intermittent versus continuous energy restriction on weight loss and cardiometabolic outcomes: a systematic review and meta-analysis of randomized controlled trials. J Transl med [internet].

[6] Mattson MP, Moehl K, Ghena N, Schmaedick M, Cheng A (2018). Intermittent metabolic switching, neuroplasticity and brain health. Nat Rev Neurosci [Internet].

[7] Nencioni A, Caffa I, Cortellino S, Longo VD (2018). Fasting and cancer: molecular mechanisms and clinical application. Nat Rev Cancer [Internet].

[8] Meynet O, Ricci JE (2014). Caloric restriction and cancer: molecular mechanisms and clinical implications. Trends Mol Med.

[9] Speakman JR, Mitchell SE (2011). Caloric restriction. Mol Aspects Med [Internet].

[10] Wei M, Brandhorst S, Shelehchi M, Mirzaei H, Cheng CW, Budniak J, et al. Fasting-mimicking diet and markers/risk factors for aging, diabetes, cancer, and cardiovascular disease. Sci Transl Med. 2017;9(377):1–12. 10.1126/scitranslmed.aai8700 PMC6816332 28202779

[11] Crow S, Eisenberg ME, Story M, Neumark-Sztainer D (2008). Suicidal behavior in adolescents: relationship to weight status, weight control behaviors, and body dissatisfaction. Int J Eat Disord.

[12] Zuromski KL, Witte TK (2015). Fasting and acquired capability for suicide: a test of the interpersonal-psychological theory of suicide in an undergraduate sample. Psychiatry Res [Internet].

[13] Bakken NW, Gunter WD (2012). Self-cutting and suicidal ideation among adolescents: gender differences in the causes and correlates of self-injury. Deviant Behav.

[14] Hilt LM, Nock MK, Lloyd-Richardson EE, Prinstein MJ (2008). Longitudinal study of nonsuicidal self-injury among Young adolescents rates, correlates, and preliminary test of an interpersonal model. J Early Adolesc.

[15] Lloyd EC, Haase AM, Zerwas S, Micali N (2020). Anxiety disorders predict fasting to control weight: a longitudinal large cohort study of adolescents. Eur Eat Disord Rev.

[16] Treasure J, Cardi V, Kan C. Eating in eating disorders. Eur Eat Disord Rev. 2012;20(1):e42–e49. 10.1002/erv.1090 21275008

[17] Stice E, Davis K, Miller NP, Marti CN (2008). Fasting increases risk for onset of binge eating and bulimic pathology: a 5-year prospective study. J Abnorm Psychol.

[18] Schaumberg K, Anderson D (2016). Dietary restraint and weight loss as risk factors for eating pathology. Eat Behav [Internet].

[19] Striegel-Moore RH, Rosselli F, Perrin N, DeBar L, Wilson GT, May A, Kraemer HC (2009). Gender difference in the prevalence of eating disorder symptoms. Int J Eat Disord.

[20] Lavender JM, Brown TA, Murray SB (2017). Men, muscles, and eating disorders: an overview of traditional and muscularity-oriented disordered eating. Curr Psychiatry Rep.

[21] Nagata JM, Brown TA, Lavender JM, Murray SB (2019). Emerging trends in eating disorders among adolescent boys: muscles, macronutrients, and biohacking. Lancet Child Adolesc Heal [Internet].

[22] Nagata JM, Domingue BW, Darmstadt GL, Weber AM, Meausoone V, Cislaghi B (2020). Gender norms and weight control behaviors in U.S. adolescents: a prospective cohort study (1994–2002). J Adolesc heal [internet].

[23] Ward ZJ, Rodriguez P, Wright DR, Austin SB, Long MW (2019). Estimation of eating disorders prevalence by age and associations with mortality in a simulated nationally representative US cohort. JAMA Netw Open.

[24] Volpe U, Tortorella A, Manchia M, Monteleone AM, Albert U, Monteleone P (2016). Eating disorders: what age at onset?. Psychiatry Res.

[25] Neumark-Sztainer D, Wall M, Larson NI, Eisenberg ME, Loth K (2011). Dieting and disordered eating behaviors from adolescence to young adulthood: findings from a 10-year longitudinal study. J Am Diet Assoc [Internet].

[26] Bucchianeri MM, Arikian AJ, Hannan PJ, Eisenberg ME, Neumark-Sztainer D (2013). Body dissatisfaction from adolescence to young adulthood: findings from a 10-year longitudinal study. Body Image [Internet].

[27] Eisenberg D, Hunt J, Speer N (2013). Mental health in american colleges and universities: variation across student subgroups and across campuses. J Nerv Ment Dis.

[28] Arria AM, Caldeira KM, Allen HK, Bugbee BA, Vincent KB, Grady KEO (2017). Prevalence and incidence of drug use among college students: an 8-year longitudinal analysis. Am J Drug Alcohol Abuse.

[29] Schulenberg JE, Johnston LD, O’Malley PM, Bachman JG, Miech RA, Patrick ME. Monitoring the Future: College Students & Adults Ages 19–60 [Internet]. 2019. Available from: http://monitoringthefuture.org/pubs.html#monographs

[30] Udo T, Grilo CM (2018). Psychiatric and medical correlates of DSM-5 eating disorders in a nationally representative sample of adults in the United States. Int J Eat Disord.

[31] Goel NJ, Sadeh-Sharvit S, Flatt RE, Trockel M, Balantekin KN, Fitzsimmons-Craft EE, et al. Correlates of suicidal ideation in college women with eating disorders. Int J Eat Disord. 2018;51(6):579–84. 10.1002/eat.22865. 10.1002/eat.22865 PMC6002903 29626350

[32] Lipson SK, Sonneville KR. Understanding suicide risk and eating disorders in college student populations: results from a national study. Int J Eat Disord. 2019;53(2):1–10. 10.1002/eat.23188 31639232

[33] Eisenberg D, Nicklett EJ, Roeder K, Kirz NE (2011). Eating disorder symptoms among college students: prevalence, persistence, correlates, and treatment-seeking. J Am Coll Heal.

[34] Gross JJ (1998). The emerging field of emotion regulation: an integrative review. Rev Gen Psychol.

[35] Aldao A, Nolen-Hoeksema S, Schweizer S (2010). Emotion-regulation strategies across psychopathology: a meta-analytic review. Clin Psychol rev [internet].

[36] Mallorquí-Bagué N, Vintró-Alcaraz C, Sánchez I, Riesco N, Agüera Z, Granero R, Jiménez-Múrcia S, Menchón JM, Treasure J, Fernández-Aranda F (2018). Emotion regulation as a Transdiagnostic feature among eating disorders: cross-sectional and longitudinal approach. Eur Eat Disord Rev.

[37] Eisenberg D, Lipson SK (2020). Healthy minds network [internet].

[38] Fairburn CG, Beglin S, Fairburn CG (2008). Eating disorder examination questionnaire. Cognitive behavior therapy and eating disorders.

[39] Lavender JM, De Young KP, Anderson DA (2010). Eating disorder examination questionnaire (EDE-Q): norms for undergraduate men. Eat Behav [Internet].

[40] Nagata JM, Capriotti MR, Murray SB, Compte EJ, Griffiths S, Bibbins-Domingo K, Obedin-Maliver J, Flentje A, Lubensky ME, Lunn MR (2020). Community norms for the eating disorder examination questionnaire among cisgender gay men. Eur Eat Disord Rev.

[41] Nagata JM, Murray SB, Compte EJ, Pak EH, Schauer R, Flentje A, et al. Community norms for the eating disorder examination questionnaire (EDE-Q) among cisgender bisexual plus women and men. Eat Weight Disord. 2020;3 Available from: https://doi.org/10.1007/s40519-020-01070-8. 10.1007/s40519-020-01070-8 PMC8437880 33270173

[42] Gollust SE, Eisenberg D, Golberstein E (2008). Prevalence and correlates of self-injury among university students. J Am Coll Heal [Internet].

[43] Serras A, Saules KK, Cranford JA, Eisenberg D (2010). Self-injury, substance use, and associated risk factors in a multi-campus probability sample of college students. Psychol Addict Behav.

[44] Kroenke K, Spitzer RL, Williams JBW (2001). The PHQ-9: validity of a brief depression severity measure. J Gen Intern Med.

[45] Spitzer RL, Kroenke K, Williams JBW, Group PHQPCS (1999). Validation and utility of a self-report version of PRIME-MD: the PHQ primary care study. JAMA..

[46] Spitzer RL, Kroenke K, Williams JBW, Löwe B (2006). A brief measure for assessing generalized anxiety disorder: the GAD-7. Arch Intern Med.

[47] Morgan JF, Reid F, Lacey JH (1999). The SCOFF questionnaire: assessment of a new screening tool for eating disorders. Br Med J.

[48] Luck AJ, Morgan JF, Reid F, O’Brien A, Brunton J, Price C (2002). The SCOFF questionnaire and clinical interview for eating disorders in general practice: comparative study. Br Med J.

[49] Maguen S, Hebenstreit C, Li Y, Dinh JV, Donalson R, Dalton S (2018). Screen for disordered eating: improving the accuracy of eating disorder screening in primary care. Gen Hosp psychiatry [internet].

[50] Lipson SK, Lattie EG, Eisenberg D (2019). Increased rates of mental health service utilization by U.S. college students: 10-year population-level trends (2007-2017). Psychiatr Serv.

[51] Lipson SK, Kern A, Eisenberg D, Breland-Noble AM (2018). Mental health disparities among college students of color. J Adolesc Health.

[52] Nagata JM, Garber AK, Tabler J, Murray SB, Vittinghoff E, Bibbins-Domingo K (2018). Disordered eating behaviors and cardiometabolic risk among young adults with overweight or obesity. Int J Eat Disord.

[53] StataCorp LLC (2020). Stata 15 [Internet].

[54] Nagata JM, Ganson KT, Austin SB (2020). Emerging trends in eating disorders among sexual and gender minorities. Curr Opin Psychiatry [Internet].

[55] Friedman MR, Dodge B, Schick V, Herbenick D, Hubach RD, Bowling J, Goncalves G, Krier S, Reece M (2014). From bias to bisexual health disparities: attitudes toward bisexual men and women in the United States. LGBT Heal.

[56] Beccia AL, Baek J, Jesdale WM, Austin SB, Forrester S, Curtin C (2019). Risk of disordered eating at the intersection of gender and racial/ethnic identity among U.S. high school students. Eat Behav [internet].

[57] Rodgers RF, Berry R, Franko DL. Eating disorders in ethnic minorities: an update. Curr Psychiatry Rep. 2018;20(10):1–11. 10.1007/s11920-018-0938-3 30155577

[58] Tabler J, Utz RL (2015). The influence of adolescent eating disorders or disordered eating behaviors on socioeconomic achievement in early adulthood. Int J Eat Disord.

[59] Mulders-Jones B, Mitchison D, Girosi F, Hay P (2017). Socioeconomic correlates of eating disorder symptoms in an Australian population-based sample. PLoS One.

[60] Mitchison D, Hay PJ (2014). The epidemiology of eating disorders: genetic, environmental, and societal factors. Clin Epidemiol.

[61] Murray SB, Nagata JM, Griffiths S, Calzo JP, Brown TA, Mitchison D, Blashill AJ, Mond JM (2017). The enigma of male eating disorders: a critical review and synthesis. Clin Psychol Rev [Internet].

[62] Bentley C, Mond J, Rodgers B (2014). Sex differences in psychosocial impairment associated with eating-disordered behavior: what if there aren’t any?. Eat Behav [Internet].

[63] Bentley C, Gratwick-Sarll K, Harrison C, Mond J (2015). Sex differences in psychosocial impairment associated with eating disorder features in adolescents: a school-based study. Int J Eat Disord.

[64] Solmi M, Veronese N, Sergi G, Luchini C, Favaro A, Santonastaso P, Vancampfort D, Correll CU, Ussher M, Thapa-Chhetri N, Fornaro M, Stubbs B (2016). The association between smoking prevalence and eating disorders: a systematic review and meta-analysis. Addiction..

[65] Bruening AB, Perez M, Ohrt TK. Exploring weight control as motivation for illicit stimulant use, Eat Behav [internet]. 2018;30(June):72–5 Available from: https://doi.org/10.1016/j.eatbeh.2018.06.002. 10.1016/j.eatbeh.2018.06.002 29886378

[66] Jeffers AJ, Benotsch EG, Koester S (2013). Misuse of prescription stimulants for weight loss, psychosocial variables, and eating disordered behaviors. Appetite [Internet].

[67] Jeffers AJ, Benotsch EG (2014). Non-medical use of prescription stimulants for weight loss, disordered eating, and body image. Eat Behav [Internet].

[68] Ganson KT, Murray SB, Nagata JM. Associations between eating disorders and illicit drug use among college students. Int J Eat Disord. 2021;(February):1–8. 10.1002/eat.23493 33638571

[69] Nagata JM, Ganson KT, Griffiths S, Mitchison D, Garber AK, Vittinghoff E, et al. Prevalence and correlates of muscle-enhancing behaviors among adolescents and young adults in the United States. Int J Adolesc Med Health. 2020. 10.1515/ijamh-2020-0001 PMC9972881 32549173

[70] Lupi M, Martinotti G, Di Giannantonio M (2017). Drunkorexia: an emerging trend in young adults. Eat Weight Disord.

[71] Thompson-Memmer C, Glassman T, Diehr A (2019). Drunkorexia: a new term and diagnostic criteria. J Am Coll Heal [internet].

[72] Anderson CB, Bulik CM (2004). Gender differences in compensatory behaviors, weight and shape salience, and drive for thinness. Eat Behav.

[73] Mitchison D, Mond J. Epidemiology of eating disorders, eating disordered behaviour, and body image disturbance in males: A narrative review. J Eat Disord [Internet], 2015. 3(1):1–9 Available from: ??? 10.1186/s40337-015-0058-y PMC4940910 27408719

[74] Milton AC, Ellis LA, Davenport TA, Burns JM, Hickie IB (2017). Comparison of self-reported telephone interviewing and web-based survey responses: findings from the second Australian Young and well National Survey. JMIR Ment Heal.

[75] Hines DA, Douglas EM, Mahmood S (2010). The effects of survey administration on disclosure rates to sensitive items among men: a comparison of an internet panel sample with a RDD telephone sample. Comput Hum Behav.

[76] Kreuter F, Presser S, Tourangeau R (2008). Social desirability bias in CATI, IVR, and web surveys: the effects of mode and question sensitivity. Public Opin Q.

