# Supplementary material for: Prevalence and demographic, substance use, and mental health correlates of fasting among U.S. college students
Source: J Eat Disord. 2021 Jul 21;9:88. doi: 10.1186/s40337-021-00443-3 (PMC8293526; doi:10.1186/s40337-021-00443-3)
Supplement: Supplementary file 1 — Additional file 1: Supplement Table 1. Demographic Characteristics and Descriptive Statistics of College Student Participants from the 2016–2020 Healthy Minds Study (N = 8255) by Gender and Survey Year. Supplement Table 2. Associations between Demographic Correlates and Survey Year and Any Fasting (≥ 1 Times) in the Past Four Weeks by Gender and BMI. Supplement Table 3. Associations between Demographic Correlates and Survey Year and Regular Fasting (≥ 13 Times) in the Past Four Weeks by Gender and BMI. Supplement Fig. 1. Prevalence (%) of Any (≥ 1 Times) and Regular (≥ 13 Times) Fasting in the Past Four Weeks among Participants with Body Mass Index < 25, by Gender and Survey Year. Supplement Fig. 2. Prevalence (%) of Any (≥ 1 Times) and Regular (≥ 13 Times) Fasting in the Past Four Weeks among Participants with Body Mass Index ≥ 25, by Gender and Survey Year. Supplement Table 4. Associations between Any Fasting (≥ 1 Times) and Regular Fasting (≥ 13 Times) in the Past Four Weeks and Substance Use and Mental Health Correlates by Gender and BMI among Participants from the Healthy Minds Study. [file 40337_2021_443_MOESM1_ESM.docx]

| Supplement Table 1  Demographic Characteristics and Descriptive Statistics of College Student Participants from the 2016-2020 Healthy Minds Study (N=8,255) by Gender and Survey Year | | | | | | | | | | |
| --- | --- | --- | --- | --- | --- | --- | --- | --- | --- | --- |
|  | 2016/2017  N=1,435 | | 2017/2018  N=4,352 | | 2018/2019  N=2,026 | | 2019/2020  N=442 | |  | |
|  | Men  (n=499) | Women  (n=936) | Men  (n=1,423) | Women  (n=2,929) | Men  (n=636) | Women  (n=1,390) | Men  (n=117) | Women  (n=325) | Men^a^ | Women^a^ |
|  | Mean ± SE / % | Mean ± SE / % | Mean ± SE / % | Mean ± SE / % | Mean ± SE / % | Mean ± SE / % | Mean ± SE / % | Mean ± SE / % | p | p |
| Demographic characteristics |  |  |  |  |  |  |  |  |  |  |
| Age | 22.41 ± 0.27 | 21.94 ± 0.18 | 22.33 ± 0.17 | 21.93 ± 0.11 | 23.85 ± 0.37 | 22.74 ± 0.24 | 23.78 ± 0.77 | 22.91 ± 0.35 | 0.020 | 0.003 |
| Body mass index (kg/m^2^) | 24.23 ± 0.21 | 23.77 ± 0.18 | 24.61 ± 0.13 | 23.73 ± 0.09 | 25.01 ± 0.31 | 24.87 ± 0.18 | 25.60 ± 0.44 | 25.22 ± 0.32 | 0.030 | < 0.001 |
| Race/ethnicity |  |  |  |  |  |  |  |  | < 0.001 | < 0.001 |
| White or Caucasian, non-Hispanic, non-Arab | 36.12 | 61.58 | 64.87 | 64.24 | 67.59 | 72.14 | 39.78 | 45.01 |  |  |
| Black or African American, non-Hispanic | 2.38 | 6.16 | 3.18 | 5.16 | 3.85 | 5.75 | 17.52 | 16.07 |  |  |
| Hispanic/Latino/a | 5.43 | 10.71 | 5.0 | 4.84 | 4.45 | 2.55 | 18.79 | 16.54 |  |  |
| Asian or Asian American | 12.52 | 8.93 | 14.36 | 14.08 | 12.66 | 9.25 | 3.67 | 4.05 |  |  |
| Arab/Middle Eastern or Arab American | 1.15 | 0.93 | 1.24 | 0.88 | 2.19 | 0.31 | 1.09 | 1.78 |  |  |
| American Indian, Alaskan Native, Native Hawaiian or Pacific Islander | 0.0 | 0.35 | 0.23 | 0.16 | 0.01 | 0.42 | 0.54 | 0.0 |  |  |
| Other race/ethnicity | 1.92 | 0.89 | 1.75 | 0.51 | 1.61 | 0.45 | 1.30 | 1.57 |  |  |
| More than 1 race/ethnicity | 13.49 | 10.44 | 9.37 | 10.11 | 7.61 | 9.12 | 17.32 | 14.99 |  |  |
| Sexual orientation |  |  |  |  |  |  |  |  | 0.518 | < 0.001 |
| Heterosexual | 88.24 | 85.97 | 85.72 | 82.18 | 85.05 | 77.75 | 86.94 | 76.69 |  |  |
| Gay or lesbian | 5.21 | 1.82 | 6.85 | 4.16 | 6.92 | 3.69 | 7.33 | 4.51 |  |  |
| Bisexual | 3.32 | 8.96 | 3.67 | 8.51 | 4.60 | 13.44 | 2.89 | 12.47 |  |  |
| Queer, questioning, or other | 3.23 | 3.25 | 3.76 | 5.15 | 3.43 | 5.12 | 2.84 | 6.32 |  |  |
| Highest parental education |  |  |  |  |  |  |  |  | 0.082 | < 0.001 |
| High school degree or less | 9.22 | 9.38 | 9.18 | 7.60 | 11.43 | 8.59 | 16.31 | 23.95 |  |  |
| Some college or more | 90.78 | 90.62 | 90.82 | 92.40 | 88.57 | 91.41 | 83.69 | 76.05 |  |  |
| Substance use correlates |  |  |  |  |  |  |  |  |  |  |
| Any cigarette use, past 30 days | 13.29 | 7.31 | 12.11 | 6.75 | 8.59 | 5.89 | 4.78 | 5.13 | 0.072 | 0.229 |
| Marijuana use, past 30 days | 31.20 | 27.16 | 26.75 | 22.64 | 19.18 | 16.08 | 21.73 | 22.23 | 0.001 | < 0.001 |
| Other illicit drug use, past 30 days | 11.33 | 10.10 | 9.83 | 4.39 | 5.30 | 3.50 | 3.76 | 6.27 | 0.003 | < 0.001 |
| Alcohol use, past 2 weeks | 66.31 | 70.67 | 71.26 | 71.96 | 59.27 | 62.14 | 49.25 | 48.88 | < 0.001 | < 0.001 |
| Mental health correlates |  |  |  |  |  |  |  |  |  |  |
| Positive depression screen, PHQ-9 | 21.61 | 28.24 | 21.25 | 25.38 | 23.57 | 27.64 | 28.02 | 33.86 | 0.223 | 0.080 |
| Positive anxiety screen, GAD-7 | 16.30 | 27.06 | 15.32 | 23.77 | 14.89 | 25.55 | 19.78 | 34.27 | 0.609 | 0.018 |
| Positive eating disorder screen, SCOFF | 11.45 | 25.53 | 14.23 | 26.57 | 14.74 | 25.49 | 17.68 | 28.86 | 0.285 | 0.540 |
| Suicidal ideation, past 12 months | 9.58 | 8.31 | 7.97 | 8.40 | 9.70 | 10.46 | 7.91 | 10.18 | 0.765 | 0.594 |
| Any non-suicidal self-injury, past 12 months | 14.92 | 18.84 | 15.84 | 20.34 | 15.93 | 21.94 | 16.70 | 21.16 | 0.766 | 0.741 |
| Any fasting (≥ 1 times), past 4 weeks | 10.30 | 17.02 | 12.67 | 17.45 | 19.14 | 18.73 | 19.81 | 27.32 | < 0.001 | < 0.001 |
| Regular fasting (≥ 13 times), past 4 weeks | 1.46 | 1.79 | 2.55 | 2.22 | 4.66 | 3.59 | 3.53 | 6.19 | 0.030 | < 0.001 |
| Note: Preconstructed nonresponse sample weighting was applied to all analyses.  ^a^Differences across survey years calculated using chi-square tests for categorical variables and one-way ANOVA for continuous variables. | | | | | | | | | | |

| Supplement Table 2  Associations between Demographic Correlates and Survey Year and Any Fasting (≥ 1 Times) in the Past Four Weeks by Gender and BMI | | | | | | | | |
| --- | --- | --- | --- | --- | --- | --- | --- | --- |
|  | BMI < 25 | | | | BMI ≥ 25 | | | |
|  | Men | *p* | Women | *p* | Men | *p* | Women | *p* |
|  | AOR (95% CI) |  | AOR (95% CI) |  | AOR (95% CI) |  | AOR (95% CI) |  |
| Age | 1.00 (0.96-1.05) | 0.793 | **0.94 (0.91-0.98)** | **0.001** | **0.97 (0.94-0.99)** | **0.016** | **0.96 (0.93-0.99)** | **0.004** |
| Race/ethnicity |  |  |  |  |  |  |  |  |
| White or Caucasian, non-Hispanic, non-Arab | Ref. | Ref. | Ref. | Ref. | Ref. | Ref. | Ref. | Ref. |
| Black or African American, non-Hispanic | 1.02 (0.38-2.73) | 0.972 | 0.60 (0.27-1.28) | 0.184 | 1.74 (0.68-4.45) | 0.244 | 1.07 (0.65-1.77) | 0.784 |
| Hispanic/Latino/a | 1.24 (0.61-2.53) | 0.546 | 1.01 (0.59-1.74) | 0.962 | 0.92 (0.36-2.37) | 0.867 | 1.16 (0.66-2.02) | 0.612 |
| Asian or Asian American | 0.64 (0.36-1.14) | 0.134 | 0.96 (0.71-1.32) | 0.839 | 0.97 (0.51-1.84) | 0.925 | 0.78 (0.45-1.36) | 0.388 |
| American Indian, Alaskan Native, Native Hawaiian or Pacific Islander | - | - | **8.24 (1.27-53.30)** | **0.027** | - | - | 0.24 (0.02-2.32) | 0.220 |
| Arab/Middle Eastern or Arab American | 2.09 (0.59-7.39) | 0.251 | 0.78 (0.25-2.44) | 0.670 | 0.89 (0.26-3.00) | 0.825 | 1.15 (0.26-5.02) | 0.855 |
| Other race/ethnicity | 1.24 (0.38-4.01) | 0.723 | 1.24 (0.37-4.14) | 0.723 | 2.07 (0.57-7.58) | 0.271 | 2.52 (0.47-13.42) | 0.280 |
| More than 1 race/ethnicity | 1.36 (0.74-2.50) | 0.316 | 1.03 (0.74-1.44) | 0.847 | **2.29 (1.23-4.26)** | **0.009** | 1.28 (0.82-1.99) | 0.270 |
| Sexual orientation |  |  |  |  |  |  |  |  |
| Heterosexual | Ref. | Ref. | Ref. | Ref. | Ref. | Ref. | Ref. | Ref. |
| Gay or lesbian | **3.63 (1.87-7.02)** | **0.001** | 0.97 (0.49-1.89) | 0.926 | 1.68 (0.69-4.05) | 0.259 | 1.92 (0.94-2.80) | 0.084 |
| Bisexual | **3.31 (1.75-6.27)** | **<0.001** | **1.78 (1.28-2.47)** | **0.001** | 2.08 (0.93-4.68) | 0.075 | **1.64 (1.07-2.50)** | **0.022** |
| Queer, questioning, or other | **2.63 (1.12-6.15)** | **0.026** | **1.85 (1.24-2.77)** | **0.003** | 2.23 (0.88-5.63) | 0.090 | 1.54 (0.83-2.87) | 0.170 |
| Parental education |  |  |  |  |  |  |  |  |
| High school degree or less | Ref. | Ref. | Ref. | Ref. | Ref. | Ref. | Ref. | Ref. |
| Some college or more | 0.73 (0.32-1.68) | 0.467 | 1.14 (0.69-1.88) | 0.618 | 0.63 (0.31-1.27) | 0.195 | **0.60 (0.36-0.99)** | **0.049** |
| Survey year | **1.43 (1.11-1.84)** | **0.005** | 1.01 (0.88-1.17) | 0.850 | **1.54 (1.28-2.00)** | **0.001** | 1.17 (0.98-1.40) | 0.076 |
| Note: Preconstructed nonresponse sample weighting was applied to all analyses.  **Boldface** indicates statistical significance (*p*<0.05).  BMI=Body mass index (kg/m^2^); AOR=Adjusted odds ratio; CI=Confidence interval | | | | | | | | |

| Supplement Table 3  Associations between Demographic Correlates and Survey Year and Regular Fasting (≥ 13 Times) in the Past Four Weeks by Gender and BMI | | | | | | | | |
| --- | --- | --- | --- | --- | --- | --- | --- | --- |
|  | BMI < 25 | | | | BMI ≥ 25 | | | |
|  | Men | *p* | Women | *p* | Men | *p* | Women | *p* |
|  | AOR (95% CI) |  | AOR (95% CI) |  | AOR (95% CI) |  | AOR (95% CI) |  |
| Age | 1.00 (0.91-1.10) | 0.960 | **0.92 (0.85-0.99)** | **0.033** | 1.02 (0.98-1.07) | 0.244 | 0.96 (0.93-1.00) | 0.083 |
| Race/ethnicity |  |  |  |  |  |  |  |  |
| White or Caucasian, non-Hispanic, non-Arab | Ref. | Ref. | Ref. | Ref. | Ref. | Ref. | Ref. | Ref. |
| Black or African American, non-Hispanic | 0.89 (0.1-7.59) | 0.918 | 0.74 (0.11-4.95) | 0.758 | 0.44 (0.09-2.16) | 0.312 | 0.71 (0.24-2.08) | 0.533 |
| Hispanic/Latino/a | 2.16 (0.63-7.41) | 0.222 | **0.13 (0.02-0.95)** | **0.045** | 1.24 (0.20-7.51) | 0.813 | 0.50 (0.17-1.51) | 0.221 |
| Asian or Asian American | 0.52 (0.16-1.73) | 0.289 | 0.87 (0.39-1.94) | 0.737 | 1.51 (0.52-4.44) | 0.447 | 0.43 (0.08-2.34) | 0.331 |
| American Indian, Alaskan Native, Native Hawaiian or Pacific Islander | - | - | - | - | - | - | - | - |
| Arab/Middle Eastern or Arab American | 4.14 (0.65-26.22) | 0.132 | - | - | 0.69 (0.08-5.68) | 0.732 | - | - |
| Other race/ethnicity | 1.81 (0.22-15.24) | 0.583 | 1.95 (0.24-15.84) | 0.530 | 0.95 (0.15-6.11) | 0.959 | - | - |
| More than 1 race/ethnicity | 0.95 (0.32-2.83) | 0.929 | 1.04 (0.45-2.41) | 0.992 | 1.65 (0.44-6.26) | 0.457 | 1.84 (0.76-4.48) | 0.177 |
| Sexual orientation |  |  |  |  |  |  |  |  |
| Heterosexual | Ref. | Ref. | Ref. | Ref. | Ref. | Ref. | Ref. | Ref. |
| Gay or lesbian | 1.71 (0.35-8.33) | 0.508 | 1.11 (0.32-3.77) | 0.869 | 1.50 (0.24-9.24) | 0.664 | 1.82 (0.54-6.30) | 0.323 |
| Bisexual | 2.51 (0.68-9.17) | 0.165 | 1.78 (0.80-4.00) | 0.160 | 1.07 (0.22-5.10) | 0.930 | 1.99 (0.82-4.84) | 0.130 |
| Queer, questioning, or other | - | - | **2.75 (1.06-7.11)** | **0.037** | 2.16 (0.60-7.80) | 0.240 | 2.73 (0.69-10.83) | 0.151 |
| Parental education |  |  |  |  |  |  |  |  |
| High school degree or less | Ref. | Ref. | Ref. | Ref. | Ref. | Ref. | Ref. | Ref. |
| Some college or more | 1.33 (0.38-4.64) | 0.654 | 0.50 (0.20-1.23) | 0.130 | 1.96 (0.62-6.18) | 0.249 | **0.32 (0.12-0.88)** | **0.027** |
| Survey year | **1.75 (1.03-3.00)** | **0.040** | 1.31 (0.87-1.96) | 0.190 | **1.57 (1.06-2.32)** | **0.025** | **1.60 (1.05-2.43)** | **0.028** |
| Note: Preconstructed nonresponse sample weighting was applied to all analyses.  **Boldface** indicates statistical significance (*p*<0.05).  BMI=Body mass index (kg/m^2^); AOR=Adjusted odds ratio; CI=Confidence interval | | | | | | | | |

Supplement Figure 1

Prevalence (%) of Any (≥ 1 Times) and Regular (≥ 13 Times) Fasting in the Past Four Weeks among Participants with Body Mass Index < 25, by Gender and Survey Year

**p* < 0.05

Supplement Figure 2

Prevalence (%) of Any (≥ 1 Times) and Regular (≥ 13 Times) Fasting in the Past Four Weeks among Participants with Body Mass Index ≥ 25, by Gender and Survey Year

**p* < 0.01

| Supplement Table 4  Associations between Any Fasting (≥ 1 Times) and Regular Fasting (≥ 13 Times) in the Past Four Weeks and Substance Use and Mental Health Correlates by Gender and BMI among Participants from the Healthy Minds Study | | | | | | | | |
| --- | --- | --- | --- | --- | --- | --- | --- | --- |
|  | BMI < 25 | | | | BMI ≥ 25 | | | |
|  | Men |  | Women |  | Men |  | Women |  |
| Any fasting | AOR^a^ (95% CI) | *p* | AOR^a^ (95% CI) | *p* | AOR^a^ (95% CI) | *p* | AOR^a^ (95% CI) | *p* |
| Any cigarette use, past 30 days | 1.32 (0.72-2.41) | 0.361 | **1.92 (1.31-2.82)** | **0.001** | 1.45 (0.85-2.47) | 0.177 | 0.98 (0.56-1.71) | 0.956 |
| Marijuana use, past 30 days | 1.25 (0.82-1.89) | 0.297 | **2.14 (1.69-2.71)** | **<0.001** | 1.49 (0.94-2.36) | 0.091 | **1.64 (1.14-2.35)** | **0.007** |
| Other illicit drug use, past 30 days | 1.02 (0.53-1.98) | 0.944 | **3.51 (2.34-5.27)** | **<0.001** | **2.00 (1.01-3.97)** | **0.048** | 1.10 (0.47 -2.57) | 0.828 |
| Alcohol use, past 2 weeks | 0.68 (0.46-1.01) | 0.055 | 1.18 (0.93-1.50) | 0.161 | 0.79 (0.52-1.23) | 0.308 | 1.09 (0.80-1.50) | 0.582 |
| Positive depression screen, PHQ-9 | **3.62 (2.42-5.42)** | **<0.001** | **4.56 (3.63-5.74)** | **<0.001** | **3.09 (2.13-4.76)** | **<0.001** | **2.82 (2.08-3.84)** | **<0.001** |
| Positive anxiety screen, GAD-7 | **3.02 (1.94-4.69)** | **<0.001** | **2.72 (2.16-3.43)** | **<0.001** | **2.35 (1.45-3.80)** | **0.001** | **2.40 (1.75-3.27)** | **<0.001** |
| Positive eating disorder screen, SCOFF | **5.68 (3.63-8.88)** | **<0.001** | **5.82 (4.62-7.32)** | **<0.001** | **3.75 (2.38-5.91)** | **<0.001** | **3.64 (2.67-4.97)** | **<0.001** |
| Suicidal ideation, past 12 months | **2.93 (1.73-4.95)** | **<0.001** | **3.13 (2.29-4.28)** | **<0.001** | **2.18 (1.19-3.99)** | **0.012** | **2.10 (1.40-3.14)** | **<0.001** |
| Any non-suicidal self-injury, past 12 months | **2.68 (1.70-4.15)** | **<0.001** | **2.94 (2.29-3.76)** | **<0.001** | **2.32 (1.45-3.71)** | **<0.001** | **2.11 (1.52-2.94)** | **<0.001** |
|  | Men |  | Women |  | Men |  | Women |  |
| Regular fasting | AOR^a^ (95% CI) | *p* | AOR^a^ (95% CI) | *p* | AOR^a^ (95% CI) | *p* | AOR^a^ (95% CI) | *p* |
| Any cigarette use, past 30 days | 0.77 (0.15-3.89) | 0.753 | 1.92 (0.69-5.32) | 0.209 | 0.70 (0.22-2.26) | 0.553 | 1.10 (0.36-3.40) | 0.866 |
| Marijuana use, past 30 days | 1.42 (0.54-3.71) | 0.474 | **2.23 (1.25-3.98)** | **0.007** | 1.50 (0.70-3.21) | 0.298 | **2.21 (1.02-4.78)** | **0.044** |
| Other illicit drug use, past 30 days | 0.25 (0.03-1.90) | 0.181 | **7.84 (3.64-16.87)** | **<0.001** | 1.41 (0.45-4.44) | 0.559 | - | - |
| Alcohol use, past 2 weeks | 1.33 (0.56-3.14) | 0.519 | 1.07 (0.61-1.88) | 0.819 | 0.58 (0.28-1.19) | 0.139 | 1.39 (0.63-3.09) | 0.414 |
| Positive depression screen, PHQ-9 | **4.08 (1.67-9.94)** | **0.002** | **6.65 (3.67-12.04)** | **<0.001** | **3.60 (1.72-7.53)** | **0.001** | **3.47 (1.65-7.30)** | **0.001** |
| Positive anxiety screen, GAD-7 | **6.48 (2.56-16.37)** | **<0.001** | **6.35 (3.44-11.73)** | **<0.001** | **2.92 (1.24-6.87)** | **0.014** | **2.25 (1.12-4.54)** | **0.023** |
| Positive eating disorder screen, SCOFF | **15.29 (6.53-35.79)** | **<0.001** | **7.92 (4.08-15.36)** | **<0.001** | **4.36 (1.95-9.72)** | **<0.001** | **4.74 (2.26-9.92)** | **<0.001** |
| Suicidal ideation, past 12 months | **5.43 (2.12-13.86)** | **<0.001** | **4.05 (2.21-7.40)** | **<0.001** | 1.77 (0.65-4.87) | 0.265 | **2.65 (1.32-6.57)** | **0.008** |
| Any non-suicidal self-injury, past 12 months | **3.95 (1.58-9.92)** | **0.003** | **3.97 (2.06-7.66)** | **<0.001** | **2.32 (1.01-5.31)** | **0.047** | 1.84 (0.84-4.00) | 0.125 |
| Note: Preconstructed nonresponse sample weighting was applied to all analyses.  **Boldface** indicates statistical significance (*p*<0.05).  BMI=Body mass index (kg/m^2^); AOR=Adjusted odds ratio; CI=Confidence interval  ^a^Adjusted for age, race/ethnicity, sexual orientation, highest parent education, and survey year. | | | | | | | | |
